# Iron metabolism in colorectal cancer: a balancing act

**DOI:** 10.1007/s13402-023-00828-3

**Published:** 2023-06-05

**Authors:** Diogo Estêvão, Miguel da Cruz-Ribeiro, Ana P. Cardoso, Ângela M. Costa, Maria J. Oliveira, Tiago L. Duarte, Tânia B. da Cruz

**Affiliations:** 1 i3S - Instituto de Investigação e Inovação em Saúde, University of Porto, Porto, Portugal; 2 ICBAS - Instituto de Ciências Biomédicas Abel Salazar, University of Porto, Porto, Portugal; 3 Laboratory of Experimental Cancer Research, Department of Human Structure and Repair, Cancer Research Institute, Ghent University, Ghent, Belgium; 4 FMUP - Faculty of Medicine, Pathology Department, University of Porto, Porto, Portugal

**Keywords:** Colorectal cancer, Iron metabolism, Hypoxia, Oxidative stress, Ferroptosis

## Abstract

**Background:**

Colorectal cancer (CRC) is the third most commonly diagnosed cancer and the second deadliest malignancy worldwide. Current dietary habits are associated with increased levels of iron and heme, both of which increase the risk of developing CRC. The harmful effects of iron overload are related to the induction of iron-mediated pro-tumorigenic pathways, including carcinogenesis and hyperproliferation. On the other hand, iron deficiency may also promote CRC development and progression by contributing to genome instability, therapy resistance, and diminished immune responses. In addition to the relevance of systemic iron levels, iron-regulatory mechanisms in the tumor microenvironment are also believed to play a significant role in CRC and to influence disease outcome. Furthermore, CRC cells are more prone to escape iron-dependent cell death (ferroptosis) than non-malignant cells due to the constitutive activation of antioxidant genes expression. There is wide evidence that inhibition of ferroptosis may contribute to the resistance of CRC to established chemotherapeutic regimens. As such, ferroptosis inducers represent promising therapeutic drugs for CRC.

**Conclusions and perspectives:**

This review addresses the complex role of iron in CRC, particularly in what concerns the consequences of iron excess or deprivation in tumor development and progression. We also dissect the regulation of cellular iron metabolism in the CRC microenvironment and emphasize the role of hypoxia and of oxidative stress (*e.g.* ferroptosis) in CRC. Finally, we underline some iron-related players as potential therapeutic targets against CRC malignancy.

## Introduction

Colorectal cancer (CRC) is the third most common and the second deadliest malignancy worldwide [1]. Disease etiology involves genetic and environmental factors, like chronic inflammation, obesity, and nutrition [2]. Pre-malignant lesions occur through a well-described sequence of genetic and epigenetic modifications [3]. The genes involved and the sequence in which they are activated result in different pathways of carcinogenesis, leading to tumors with distinct types of genetic instabilities [4]. These pre-malignant lesions can develop via the adenoma-carcinoma or the serrated neoplasia pathway. The former begins with Adenomatous Polyposis Coli (*APC*) mutation, followed by Rat Sarcoma virus (RAS) protein activation or Tumor-Protein p53 (TP53) loss of function, and accounts for 70–90% of CRC cases. The serrated neoplasia pathway is related to RAS and to proto-oncogene *c-RAF* mutations and CpG island methylation phenotype leading to microsatellite stable (MSS) or unstable (MSI) tumors [5]. CRC classification currently relies on consensus molecular subtypes (CMS) reflecting both molecular and cellular biological differences. These are CMS1 (MSI Immune), CMS2 (Canonical), CMS3 (Metabolic) and CMS4 (Mesenchymal) [6]. CMS1 tumors are hypermutated, generally MSI and have strong immune activation [7]. Whilst CMS2 and CMS3 are mainly epithelial and account for approximately 50% of colon cancers, CMS3 is characterized by marked metabolic dysregulation [8]. CMS4 has strong TGF-β activation, stromal invasion, fibrosis, and angiogenesis [7]. Additionally, this recent and sophisticated classification system considers that colon and rectal carcinomas may be infiltrated with numerous immune cells, namely macrophages and T lymphocytes, as well as fibroblasts, adipocytes, and endothelial cells, all of which form the complex CRC tumor microenvironment (TME). The cell type, density, function, localization as well as the extracellular matrix composition are known to influence the course of the disease and the response to therapy [9]. Specifically, the immune landscape of CRC is highly relevant for predicting patients’ prognosis [10, 11]. Importantly, carcinogenic mechanisms, cell recruitment, and response of CRC to therapy are all affected by the balance in the levels of essential nutrients (*e.g.* iron).

Iron is a crucial nutrient for virtually all living organisms and can be found either directly bound to proteins or as a co-factor, namely in the form of heme or iron-sulfur clusters. These iron-containing proteins are essential for several biological and cellular processes, including oxygen transport, energy production, and cellular proliferation [12]. In aqueous solution iron can exist in two main forms, ferrous (Fe^2+^) and ferric (Fe^3+^) iron, easily engaging in oxidation–reduction reactions due to its ability to donate and accept electrons [13]. The possibility of interchanging between oxidation states enables iron to engage in the Fenton reaction, generating reactive oxygen free radicals such as Hydroxyl radicals which promptly react with and damage proteins, fatty and nucleic acids [14–16]. Therefore, iron levels are finely tuned by a variety of highly regulated proteins, which act in concert to mediate iron homeostasis and avoid toxicity. Emerging evidences indicate that, in cancer, the balance between iron surplus and iron deficiency is paramount as it impacts tumorigenesis, progression, drug resistance, and immune activation and escape [17]. In CRC, even though there are some conflicting data, it is clear that a high intake of red and processed meat, iron and heme-rich substances, is associated with an increased risk of disease development and progression [18–21]. Interestingly, iron deficiency (*e.g.* anemia, iron chelation), the flipside of the coin, also displays controversial roles as it may i) limit cancer cell growth, ii) protect malignant cells from iron-dependent cell death (ferroptosis), and iii) hinder immune surveillance [16, 22–24]. In addition to the role of systemic iron levels, in CRC the mechanisms behind iron-mediated cellular processes are also known to influence the disease outcome. For example, the up-regulation of the Transferrin Receptor 1 (TfR1) (responsible for cellular iron intake) and ferritin (the cellular iron storage protein) by malignant cells seems to be essential for tumor progression [25, 26]. Tumor-associated macrophages (TAMs), displaying an anti-inflammatory profile are known to act as “iron donors”, that may either feed proliferative cancer cells or contribute to an oxidative graveyard [27–29]. In advanced CRC tumors, the hypoxic core activates hypoxia-inducible factors (*i.e. HIF*α and β) that bind to Hypoxia Responsive Elements (HREs) altering the expression of several iron metabolism genes [30]. This review addresses the role of iron in CRC, particularly regarding the consequences of iron over-sufficiency and deprivation in tumor development and progression. The article will also dissect the regulation of cellular iron metabolism in the CRC microenvironment and pinpoint some iron-related players as potential therapeutic targets against CRC malignancy.

## Systemic iron: a double-edged sword in colorectal cancer

**Fig. 1:**
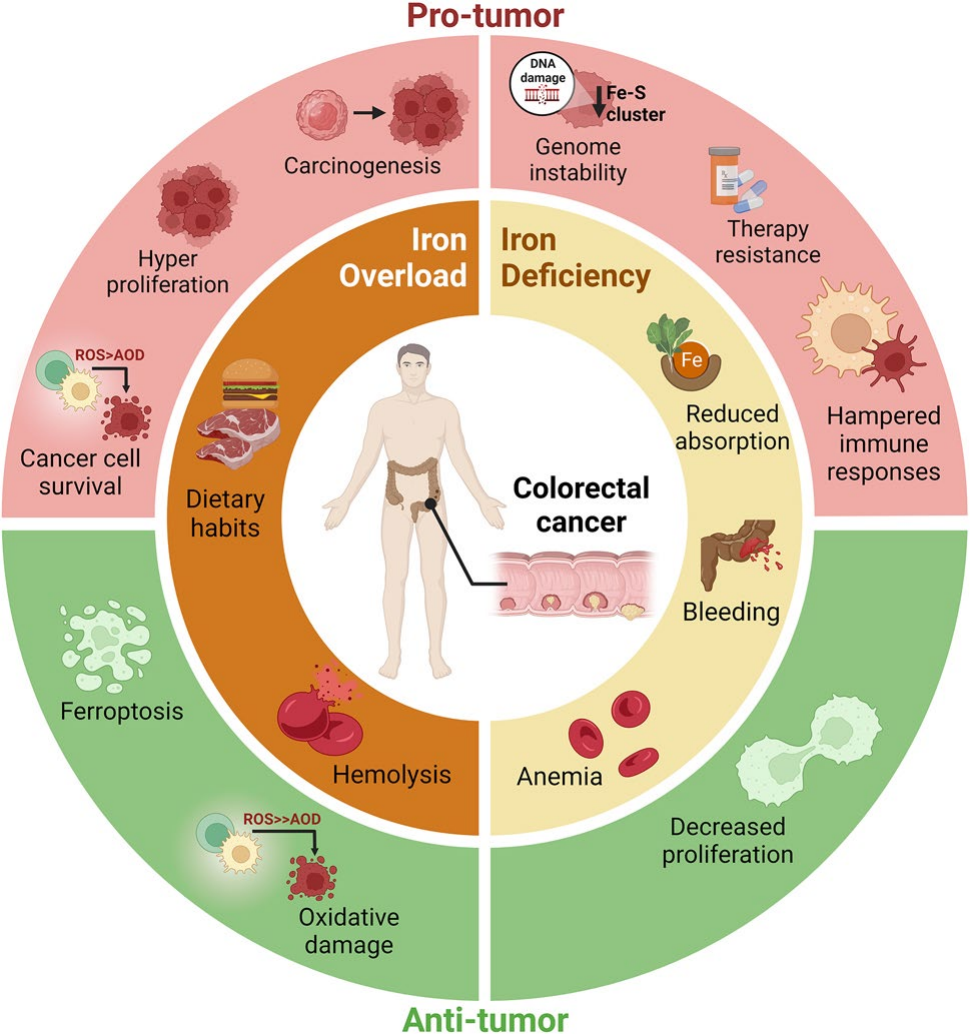
Balancing systemic iron levels is essential to prevent CRC development. Current dietary habits, particularly in the western world, are associated with increased levels of iron and heme, both of which increase the risk of developing CRC. Hemolysis, often associated with anemia, may also result in systemic iron accumulation and, consequently contribute to a high risk of CRC. The harmful effects of iron overload are related to the induction of iron-mediated pro-tumor pathways namely, WNT-signaling, AKT, MAPK, EGFR, associated with hyperproliferation, cell survival, and consequently carcinogenesis. Interestingly, high levels of iron may also contribute to anti-tumor processes such as oxidative damage of cancer cells and death by ferroptosis. In turn, iron deficiency, associated with reduced absorption, bleeding or anemia, may also assist in CRC containment since iron is an essential nutrient for cancer cell proliferation. On the other hand, iron deficiency, by contributing to genome instability, therapy resistance, and diminished immune responses, may also promote CRC progression. This complexity highlights the need to study the iron metabolism of CRC patients, at different stages of the disease, when considering iron supplementation or chelation therapeutics

The average of the total amount of iron in the human body is approximately 3 g, with 1 to 4 mg of the metal being taken up daily from the diet through duodenum enterocytes [14]. Dietary iron is then released into the plasma to compensate for organism losses, mainly by desquamation of epithelia and mucosa surfaces, bleeding, sweating, and urinary excretion [31]. Dietary habits, particularly the disproportionate consumption of heme iron-rich (red and processed meat) and non-heme iron-containing foods (seeds, nuts, grains, and dark green leafy vegetables) may increase systemic iron levels [32]. A high intake of iron-rich food, particularly of heme-containing meals, such as red and processed meat, has been reported by the International Agency for Research on Cancer (IARC) to be associated with CRC risk (Fig. 1) [33, 34]. This is supported by several epidemiological studies namely, Chao et al*.* who reported that long-term consumption of red meat was associated with higher rectal cancer risk [35], a cohort study in the Netherlands that uncovered a direct association between high heme intake and the risk of CRC containing *KRAS* activating and *APC* overall mutations (G > A) [36], one branch examination of the Iowa Women’s Health Study including 34,708 postmenopausal women, aged 55–69 years, which evidenced that increased dietary heme associates with a higher risk of colon cancer in alcohol-consuming women [37], and one analysis of 2719 CRC cases from the NIH-AARP Diet and Health Study (USA) whose conclusion was that the unrestrained intake of processed meat is associated with a higher risk of rectal cancer than of colon cancer, most likely due to heme iron, nitrate/nitrite, and heterocyclic amines [38]. Apart from epidemiological studies, numerous meta-analyses aimed at clarifying the impact of dietary heme and iron on CRC risk. Herein we highlight the following: a study that revised 59 epidemiological reports from 1995 to 2012 [39], a study that reviewed 20 human reports from 1996–2012 [40], and another study that included 14 cohort and 15 case–control studies up to 2017 [41]. Despite some discrepancies likely due to population specificities or lack of power, all of these reports concluded that excessive iron and heme consumption, mainly from red meat, is positively associated with cancer risk, correlating with a higher incidence of both colon and rectal cancer. Of note, a recent study by Aglago and colleagues explored the association between the dietary intake of total, heme, and non-heme iron and CRC risk in men and women resorting to a European prospective cohort with over 6000 CRC cases. According to the authors, in men, total iron intake was not associated with CRC risk and heme iron intake revealed a statistically non-significant association with CRC risk while non-heme iron intake was inversely correlated with the incidence of CRC. In women, however, the intake of total dietary, heme, or non-heme iron were not linked to an increased risk of CRC [42].The molecular basis of such association has not been fully elucidated, but one possible mechanism is the hypomethylation of oncogenes. Upon iron treatment, in vitro, colon cancer Caco-2 cells hyperproliferate and were shown to have over 50 hypomethylated genes from the EGFR, MAPK, and Akt tumorigenic pathways [43]. Iron excess may also exacerbate colorectal tumorigenesis by promoting Wnt signaling on CRC cells bearing-*APC* mutations [44]. Additionally, heme iron is a potential pro-oxidant capable of generating reactive oxygen species (ROS), acting as a nitrosating agent, and consequently, increasing lipid peroxidation and carcinogenic N-nitroso compounds [21, 33, 39]. In line with the evidence presented above, the modulation of elevated iron/heme levels with iron chelators could represent a potential therapeutic approach against CRC. Indeed, a highly selective tumor-targeted iron-chelating molecule, Quilamine HQ1-44, which inhibits DNA synthesis and cell proliferation, reduced HCT116 cell and tumor growth in vitro and in vivo, respectively [45]. More recently, desferioxamine (DFO), a clinically approved iron chelator, was shown to decrease the proliferation of HCT116 but not of LoVo cells and to modulate the global histone methylation status of both CRC cell lines [46]. Interestingly, a number of studies have provided compelling evidence that curcumin, a yellow spice with iron chelating properties, inhibits HCT-15, HT-29, and LoVo cell proliferation, enhances ROS levels, decreases mitochondrial membrane potential, activates caspase-3 and -9, promotes DNA fragmentation, chromatin condensation, and cell nuclear shrinkage, leading to apoptosis in a dose-dependent manner [47–49]. Moreover, experimental data obtained from murine models identified curcumin as a radio-sensitization mediator. The efficacy of curcumin in reducing tumor growth when combined with ionizing radiation is likely associated with ROS production, down-regulation of pre-mRNA processing factor 4, and suppression of the NF-kB pathway [50, 51]. These promising findings led to the development of several clinical trials to test the effect of oral curcumin supplementation on CRC patients. Although these studies indicate a potential role of curcumin in CRC prevention and treatment, a better knowledge of its properties and mode of action is still required. For example, it remains to be determined whether the above mentioned effects of curcumin are indeed related with iron chelation. Anyhow, the usage of iron chelators alone or in combination with chemotherapeutic agents, together with innovative technologies such as nanotechnology, targeted and cell therapies, and biochemical engineering, remains to be explored as potential therapeutic applications against CRC.

Interestingly, the most common hematological condition in CRC patients is iron deficiency, occurring in about 60% of diagnosed individuals [16, 52]. Iron deficiency is associated with CRC risk (Fig. 1) [53] and may benefit disease progression as it hampers immune cell functions, thus compromising tumor surveillance, cytokine production, oxidative defense, response to treatment, and the expression/activation of cancer-associated genes (*i.e. HIF*, *VEGF*, *NRF2*) [16, 24, 54–58]. It was also reported that iron deficiency is associated with lower disease-free survival rates, particularly in individuals with advanced right-side tumors or with T3N0M0 stage colon cancer [59]. It occurs due to tumor-induced bleeding (particularly in the rectum), chronic inflammation, and reduced iron absorption [22, 26], and is often associated with anemia, as more than 70% of CRC patients display decreased hemoglobin and hematocrit levels [26, 60]. A study performed with a cohort of 339 CRC patients indicated that, at diagnosis, 48.1% of the individuals presented iron deficiency of which 56.4% were anemic. Of note, anemia developed in 50% of the patients with colon cancer, compared to only 20% in rectal cancer cases [22]. Another study reported that in a cohort of 366 CRC patients, approximately 50% presented iron deficiency and, about 30% of the individuals had anemia [23]. The development of anemia, either due to low iron levels or inflammation, is associated with a major iron metabolism orchestrator, hepcidin [61]. This hormone is known to bind the cellular iron exporter Ferroportin 1 (FPN1) promoting its degradation, which results in decreased serum iron levels [62]. In a study performed by Ward et al*.*, hepcidin mRNA expression was detected in 34% of the samples from a small cohort of CRC patients, but not in the adjacent normal colon mucosa [63]. Another study found that CRC patients have a 2.9-fold decrease in hepcidin mRNA levels in tumor tissues, while serum hepcidin levels were within the range of controls. Despite this, the authors speculated that in CRC patients’ serum hepcidin levels may be inappropriately high given their degree of iron restriction, which may reduce duodenal iron absorption, increasing the exposure of the colonic adenocarcinoma to iron. The increased hepcidin may be attributed to the mild inflammation of CRC patients, which was evidenced by significantly increased tumor IL-6 mRNA and serum C-Reactive Protein levels [64]. In the future it would critical to clarify the role of both systemic and locally produced hepcidin in CRC, by comparing: i) healthy individuals with CRC patients and ii) samples from tumors paired with adjacent normal mucosa, in larger cohorts.

Apart from anemia, other parameters used to assess iron stores were also described as possible biomarkers for the relationship between iron deficiency and CRC progression. A cohort study by Herrinton et al*.* showed that low levels of transferrin saturation, an indirect measure of tissue iron levels, were associated with a high risk for the development of colon and rectal carcinoma in men [65]. Three nested case studies revealed that low levels of serum ferritin are associated with an elevated risk of developing colon but not rectal cancer [66–68]. In these studies, however, contradictory results were obtained for serum iron, transferrin saturation, and total iron binding capacity, with two studies showing no association with CRC risk [66, 67], while a third study presented an inverse association of these systemic iron indicators with colon cancer risk [68]. Strikingly, a report on the relationship between preoperative serum ferritin levels and CRC patient survival showed that individuals with either aberrantly low or high ferritin levels in the serum had a shorter survival rate when compared to those with normal values [69]. As such, the epidemiological studies seem to suggest that normal ferritin levels in the blood (women 11–148 ng/mL; men 30–215 ng/mL) are associated with a better outcome in patients with advanced CRC [69]. Another cohort study with 965 participants demonstrated that the intake of either low (< 11.6 mg/day) or excessive (> 27.3 mg/day) iron levels increased the risk of developing adenomatous polyps, a lesion that precedes CRC [70]. These data show that, despite the existence of controversial results in the literature, iron unbalance in CRC is a potential promotor of both disease development and progression.

## The Yin Yang of cellular iron metabolism in colorectal cancer

One of the most important hallmarks of cancer is the fact that cancer cells not only proliferate rapidly but also may divide uncontrolably [71]. This higher proliferation rate is linked with an increased biosynthesis of nucleic acids and proteins and consequently a higher demand for energy [72]. To sustain and maintain their high metabolic states, malignant cells require high levels of iron [73]. This is achieved by several strategies that involve the modulation of iron acquisition, trafficking, and storage (Table 1) [84]. Brookes and colleagues were among the first to demonstrate increased expression of iron uptake proteins and increased iron storage in CRC in comparison with normal colorectal mucosa, with CRC samples presenting a higher expression of duodenal cytochrome B ferrireductase (DCYTB), divalent metal transporter-1 (DMT1), and TfR1, along with increased iron content [74]. Cui et al*.* also reported an increase of TfR1 in primary CRC tissues when compared to paired controls. Further supporting the significance of high TfR1 expression in CRC, the authors down-regulated TfR1 and subsequently demonstrated a decrease in the proliferation of different CRC cells in vitro and a suppression of tumor growth in mice [75]. TfR1 expression was also associated with tumor pathological features, with poorly differentiated tumors presenting lower TfR1 expression (32.1%) when compared to well differentiated tumors (70%). In addition, CRC patients without lymph node invasion present high TfR1 levels, smaller tumors (< 5 cm) and higher TfR1 expression when compared to larger tumors (63% versus 50%, respectively) [75]. Additionally, TfR1 positive expression is associated with a longer survival time of CRC patients, when compared with TfR1-negative patients (72.06 ± 4.26 months versus 56.05 ± 5.29 months) [75]. The regulation of TfR1 occurs at several levels, one of which is through Iron Regulatory Proteins (IRP) 1 and 2. These post-transcription regulators bind to mRNA iron-responsive elements (IREs) and stabilize the *TfR1* transcript, increasing its protein expression levels [85]. In CRC samples, *IRP2* mRNA is overexpressed and correlates positively with TfR1 mRNA when compared to normal adjacent mucosa. Accordingly, *IRP2* knockdown reduced *TfR1* mRNA and protein in RKO cells [76]. A positive correlation between *IRP2* mRNA expression and *BRAF* mutations (TCGA analysis) was also reported [76]. The latter was experimentally validated by inhibiting the BRAF downstream molecule MEK with Trametinib, in RKO and HT29 cells (*BRAF* mutated), resulting in the down-regulation of IRP2 and TfR1 when compared to untreated cells [76, 86]. Moreover, the non-coding RNA miR-107, which is down-regulated in several CRC cell lines (LoVo, SW480, HT29, DLD-1, SW620) and in patient CRC tissue, was proposed to negatively regulate TfR1 expression, affecting the proliferation, invasion, and migration of CRC cells [87]. While further studies must be performed to clarify the mechanism underlying miR-107-mediated TfR1 regulation, it seems clear that this microRNA is a promising molecular target in CRC.

**Table 1:** The ups and downs of iron metabolism players in colorectal cancer

Biological process	Protein	Function	Localization	Cell type	Experimental model	Status	Ref
Iron import	TfR-1	Binding and endocytosis of Tf-Fe^3+^	Plasma membrane	Cancer cells	HT-29	Up-regulated (mRNA)	[25]
				ND	CRC human tissue vs normal adj. mucosa	Up-regulated	﻿[74–76]
				TILs		Up-regulated	[77]
	DMT-1	Fe^2+^ iron importer	Endosomes; Plasma membrane	Cancer cells	HT-29	Up-regulated (mRNA)	[25]
				ND	CRC human tissue vs normal adj. mucosa	Up-regulated	[74]
	DCTYB	duodenal cytochrome B ferrireductase	Plasma membrane	ND	CRC human tissue vs normal adj. mucosa	Up-regulated	[74]
	HO-1	Heme-oxygenase 1	Endoplasmic reticulum	ND	CRC human samples	Up-regulated	[78]
						Down-regulated (mRNA)	[79]
				TAMs		Up-regulated	[80]
Iron storage	Ferritin-H	Ferroxidase; Iron reservoir	Cytosol	ND	CRC human tissue vs normal adj. mucosa	Down-regulated (mRNA)	[25]
						No differences	[74]
Iron export	FPN-1	Iron exporter FPN-1 degradation	Plasma membrane	Cancer cells	HT-29	Up-regulated	[25]
				ND	CRC human samples	Up-regulated (Intracellular)	[74]
					CRC mouse tissue vs normal adj. mucosa	Not expressed	[81]
	Hepcidin	FPN1 regulator	Secreted	ND	CRC human tissues	Up-regulated	[63]
						Down-regulated (mRNA)	[64]
Iron chelator	NGAL/LCN2	Siderophore chelator	Secreted	ND	CRC human tissue vs normal adj. mucosa	Up-regulated	[82]
				Cancer cells	CW2, HCT116, SW480, LOVO, LS513, HT29	Up-regulated	[83]

*ND* not determined

**Fig. 2:**
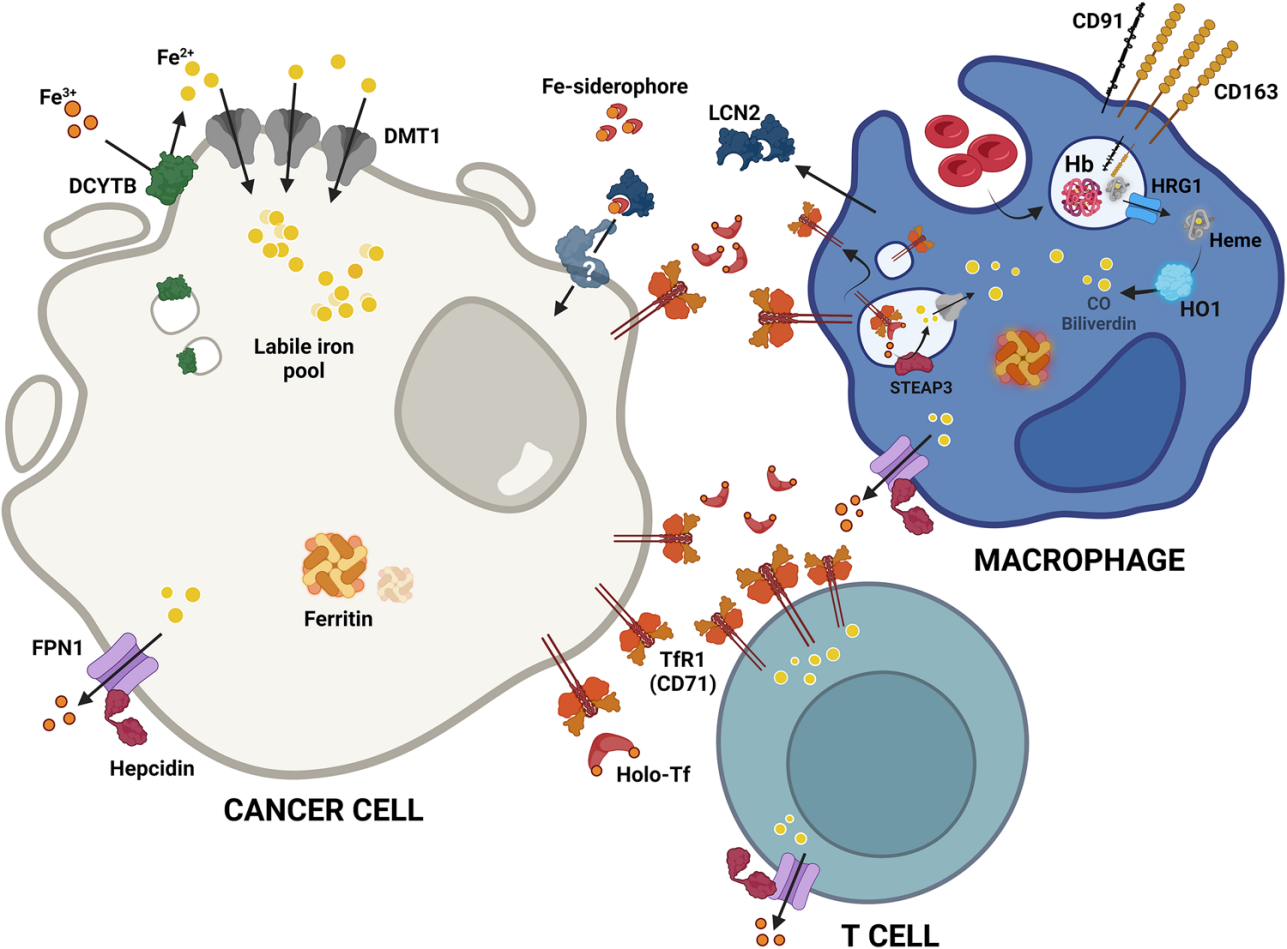
Summary of the iron metabolism players expression in the CRC microenvironment. At CRC microenvironment, cancer cells, macrophages and T lymphocytes compete for iron. Highly proliferative cancer cells increase their intracellular labile iron pool by different mechanisms namely, increase of divalent metal transporter 1 (DMT1) to facilitate the acquisition of ferrous iron (Fe^2+^), previously reduced by duodenal cytochrome b reductase (DCYTB), increase of transferrin receptor 1 (TfR1) to outcompete with other cells for transferrin-bound iron (Holo-Tf), and uptake of lipocalin 2 (LCN2), a siderophore binding protein produced by macrophages in the presence of IFNγ, TNFα and IL-17. Interestingly, CRC cells present no alterations in ferroportin (FPN1) and its regulator (hepcidin), nor in the iron storage protein, ferritin. The anti-inflammatory macrophages (M2-like), present at the CRC microenvironment, express high levels of the hemoglobin (Hb) scavenger CD163 and of TfR1. These phagocytes, upon performing erythrophagocytosis, also access Hb and heme that is then degraded by heme-oxygenase 1 (HO1) into Fe^2+^, biliverdin and carbon monoxide (CO). On their turn, T cells frequently upregulate TfR1 expression to augment their intracellular iron levels required for the sustainance of immune response. Fe^3+^, Ferric iron; Fe-siderophore, iron-loaded siderophore; CD91, heme scavenger; HRG1, heme transporter 1; STEAP3, six-transmembrane epithelial antigen of prostate 3

Recently, an alternative mechanism of iron transfer within the TME has been proposed (Fig. 2). It relies on Lipocalin-2 (LCN2), a protein that was first identified as a defense mechanism during innate immune responses, since it binds iron-loaded siderophores, sequestering iron and hampering pathogen survival [88, 89]. Interestingly, *LCN2* mRNA and protein expression were up-regulated in a cohort of 80 CRC samples, when compared with the adjacent normal tissue [82]. By modulating LCN2 in CRC cell lines (SW620 and RKO), Feng et al*.* have shown that LCN2 impacts cell proliferation, epithelial-to-mesenchymal transition (EMT), invasion, and metastasis in CRC [83]. This was also observed in vivo, since BALB/c nude mice injected with LCN2-CRC expressing cells exhibited smaller tumors, in both volume and weight, as well as a decreased ki-67 expression, when compared to mice injected with CRC cells with *LCN2* knockdown [83]. Additionally, the authors verified a decrease in E-cadherin and an increase of vimentin in *LCN2*-knockdown mouse xenograft tissue when compared to control xenografts. These findings indicate that LCN2 regulates crucial markers of phenotypic plasticity of CRC [83]. Nevertheless, more studies are required to determine the exact role of LCN2 in tumor growth and invasion.

In addition to iron uptake, cancer cells may modulate the expression of their key iron storage protein, ferritin [90]. In a small collection of paraffin-embedded tissue samples, ferritin was significantly less expressed in low- and high-grade dysplastic adenomas when compared to normal mucosa, but presented no differences in CRC adenocarcinoma samples compared to matched controls [74]. Another study comprising only 21 samples, indicated that the mRNA levels of ferritin Heavy chain (*FtH*) were down-regulated in CRC samples when compared to controls [25]. These contradictory data may be biased by the number of cases studied in the above-mentioned reports, which should be further investigated.

Progression of CRC is also associated with a reduction in the levels of the iron export machinery components. Down-regulation of hephaestin (HEPH), a transmembrane copper ferroxidase required for effective iron transport from intestinal enterocytes into the circulation, results in intestinal iron accumulation [91]. HEPH is regulated by caudal‐related homeobox 2 (CDX2), a transcription factor that is repressed in poorly differentiated human colon carcinomas [92]. Interestingly, down-regulation of the CDX2/HEPH axis by cancer associated fibroblasts-derived exosomal miR-24-3p was shown to increase chemoresistance of colon cancer cells [93]. The iron exporter protein FPN1 was reported to be more expressed in CRC samples. However, FPN1 appeared to locate intracellularly, implying that its up-regulation translates into non-functional protein. Nonetheless, poorly differentiated cells at the tumor invasive front presented low levels of FPN1 expression [74]. Using a sporadic CRC mouse model, Schwartz and colleagues showed that FPN1 while abundant in the adjacent mucosa is not expressed at the tumor tissue [81]. Moreover, the deletion of *Slc40a1*, encoding FPN1, in the colon epithelium resulted in enhanced tumor burden. The regulation of FPN1 was attributed to an intra-tumoral response to hypoxia which led to an up-regulation of locally produced hepcidin [81]. It would be interesting to investigate if FPN overexpression at the CRC cell membrane could reduce intracellular iron and lead to decreased cancer cell growth. Still, this approach could be rather difficult to translate into the clinical settings.

Another important player in iron metabolism is heme-oxygenase 1 (HO-1), an enzyme that degrades heme to produce carbon monoxide (CO), biliverdin, and free iron [94]. In a study with 55 CRC samples, HO-1 expression was detected in 41.8% of the cases and correlated with a better survival rate [95]. In another study with 118 CRC patient cases, HO-1 expression was significantly elevated in CRC tumor tissues when compared to paired normal non-tumoral and polyps samples. In addition, well-differentiated colon and rectal cancer tissues expressed the highest levels of HO-1 when compared to moderately/poorly-differentiated cancer samples [78]. A recent publication, in which 101 CRC cases were studied, reported that HO-1 was expressed in macrophages present at the tumor nest. Moreover, this study correlated the expression of HO-1 with a shorter disease-free survival time and higher number of lymph node metastasis [80]. However, an Australian retrospective cohort study with 50 cases showed that HO-1 mRNA expression was lower in cancer tissue when compared to adjacent mucosa and that lymphovascular invasion was significantly higher in HO-1 low-expressing patients. The authors also observed a trend for a longer overall survival in patients expressing high HO-1 levels [79]. The contribution of HO-1 to CRC carcinogenesis is also unclear. Some reports indicate that, in a functional p53 background, HO-1 has an anti-tumoral role through the induction of cell cycle arrest and apoptosis [96], while others suggest that HO-1 promotes tumor progression and metastasis by reducing the expression of intercellular adhesion molecule 1 (ICAM-1) and CXCL10 and consequently decreasing T cell-mediated cytotoxicity against CRC cells [97]. Finally, another study showed that HO-1 is essential for the protection of colonocytes against heme-mediated ROS formation and oxidative DNA damage [98]. Overall, these conflicting findings highlight, once again, the need for further investigating iron metabolism in the context of CRC.

Cancer cells also take advantage of immune cells, such as macrophages and T lymphocytes, present in the tumor milieu, to obtain iron [90]. TAMs are one of the major immune populations present at the CRC TME [27]. They mainly differentiate from monocytic precursors from the blood and are chemotactically recruited to the tumor by cancer cell-derived cytokines such as CCL2, VEGF, CCL5 and TGF-β [99]. In CRC, macrophage infiltration is variable depending on the CMS [100] and may affect disease prognosis (*i.e.* overall survival) [27]. As shown by our group and others, this may be related to the distribution of macrophage sub-populations within the TME while M1-like macrophages, essentially present in the normal adjacent mucosa, display a pro-inflamatory protective role, M2-like macrophages, mainly located at the invasive front, are a risk factor for CRC patients, harbouring anti-inflamatory and pro-tumor activities [27, 101, 102]. To meet their high iron demand, malignant cells could subvert the profile of TAMs into an anti-inflammatory state characterized by an “iron recycling” phenotype. These “iron donor” phagocytes display lower iron storage and increased iron efflux, primarily due to FPN1 up-regulation, feeding the tumor cells and supporting cancer progression [28]. Additionally, HO-1 is up-regulated in CRC TAMs, which is associated with a poor prognosis, possibly, due to the iron release from heme (Table 1) ﻿[78, 80]. Malignant cells can also hijack the macrophage erythrophagocytosis process. Upon de novo blood vessel formation at the TME, there is leakage of erythrocytes that will be readily cleared by macrophages and, subsequently, hemoglobin degradation will contribute to the increase of iron availability, possibly boosting cancer cell proliferation [103, 104]. Still, in the context of CRC, more studies are required to fully clarify the distinct macrophage populations present in the TME, as well as the role of iron deficiency in the inflammatory patterns within the CRC-specific TME.

In addition to the indisputable role of TAMs in the TME, solid tumors contain another important immune cell population, the tumor-infiltrating lymphocytes (TILs) [105–107]. In CRC, TILs are an heterogeneous population of lymphocytic B and T cells that impacts disease progression [107]. Infiltration of the tumor with cytotoxic CD8^+^ T lymphocytes is associated with a better prognosis [105]. Likewise, high levels of CD4^+^ Th1 cells in the tumor nest and invasive front are correlated with better overall survival and extended disease-free survival [107, 108]. On the other hand, T regulatory FOXP3^high^ immunosuppressive population is associated with a worse prognosis, whereas the FOXP3^low^ CD45RA^−^ pro-inflammatory subset is associated with a better prognosis [109]. T lymphocyte activation, function, and subsequent response are dependent on iron. In fact, the up-regulation of TfR1 (also known as CD71) is one of the earliest events in T cell activation [110]. In the TME, the diversion of iron into cancer cell proliferation may result in low iron availability for TILs, inhibiting T cell proliferation, and consequently hampering the action of T cells, while fueling cancer cell activity [77]. Apart from the TfR1, to our knowledge, nothing is known about the axis between T cell iron metabolism and CRC, thus representing an unexplored niche for the discovery of novel therapeutic targets and possibly biomarkers of response to therapy. Figure 2 summarizes the disordered regulation of iron metabolism in cancer cells, macrophages and T lymphocytes. It would also be very important to investigate the iron metabolism of NK and dendritic cells, as well as of other TME immune-related players, to clarify the contribution of the iron-immune axis in CRC pro- and anti-tumoral responses.

## The crosstalk of hypoxia and iron metabolism in colorectal cancer

The solid tumor milieu is a harsh hypoxic environment due to aberrant cancer cell growth and abnormal vascularization [111]. This low oxygen tension induces the stabilization of HIF transcription factors, heterodimer complexes composed by an α subunit, with three different isoforms, 1α, 2α, and 3α, and a β subunit [112, 113]. The stabilization of HIF is mediated by the inactivation of Prolyl 4-Hydroxylases (PHDs), allowing the rapid translocation of HIFα to the nucleus where it forms a heterodimer with the HIFβ subunit, and induces the activation of genes with HREs in their promotors [111–114]. Of note, apart from being oxygen-dependent, PHDs use iron as cofactors. Low iron levels may also lead to their inactivation and consequent HIF stabilization, even in normoxic conditions [112].

Intestinal epithelial cells express both HIF1α and HIF2α with non-redundant roles [115], and both are overexpressed in several tumors, including CRC [116, 117]. Previous studies have demonstrated that HIF2α is the isoform associated with the regulation of iron metabolism in a non-cancer context and CRC. In a non-cancer context, it is known that hypoxia links erythropoiesis with iron homeostasis and that the hepcidin/FPN1 axis regulates intestinal HIF2α to modulate iron absorption, through transcriptional activation of *DMT1*, *DCYTB*, and *FPN1* [118–120]. Moreover, the *HIF2α* promotor has IREs, to which IRPs 1 and 2 bind with high affinity, resulting in the regulation of various iron-related genes [121]. In a mouse model of colitis-associated colon cancer, HIF2α was shown to regulate *Dmt1* activation, leading to an increment of intracellular iron levels and consequently contributing to increased cell proliferation and tumor growth [122]. The authors demonstrated that increased iron levels were essential for colon tumor formation after HIF2α activation [122], which is particularly relevant since DMT1 is known to be overexpressed in colon cancer compared with normal tissue [123–125]. Using the same mouse model, HIF2α was demonstrated to also trigger the activation of the iron reductase six-transmembrane epithelial antigen of the prostate 4 (STEAP4), causing mitochondrial iron accumulation, elevated ROS levels, and increased tumor burden [126]. Interestingly, the link between HIF-2α and iron in CRC may not only result in induced carcinogenesis but also represent a vulnerability that could be explored to develop new therapies against CRC. The fact that HIF-2α activation up-regulates lipid and iron regulatory genes in CRC cell lines and colon tumors leading to a ferroptosis-susceptible cell state, indicates that reprogramming of both iron and lipid metabolism via HIF-2α, could induce malignant cell death contributing to tumor elimination [127, 128]. Despite the above-mentioned preponderant role of HIF2α in iron metabolism modulation, it is worth mentioning that iron regulates HIF1α protein stabilization by modulating cyclooxygenase-2 (COX-2) signaling in human colon cancer cells [129].

## Iron-associated oxidative stress and ferroptosis in colorectal cancer

The breakdown of the colonic barrier integrity, iron may assist inflammatory cells, such as neutrophils and macrophages, to produce ROS via Fenton's/Haber-Weiss reactions. In addition, immune cells also produce reactive nitrogen species (RNS). Together, ROS and RNS directly induce DNA base oxidation [130, 131]. Premutagenic oxidative DNA base lesions such as 8-hydroxy-2′-deoxyguanosine (8-OHdG) accumulate in the mucosa and may initiate colon carcinogenesis [132, 133]. ROS can also attack polyunsaturated fatty acids (PUFAs), which are incorporated in the membrane phospholipids of colon tissues, leading to the formation of endogenous lipid-derived electrophiles, including malondialdehyde (MDA) and 4-hydroxynonenal (4-HNE) [134]. At high levels, MDA and 4-HNE react with several DNA nucleosides to form adducts which, in the absence of repair, may lead to mutations [135–137]. Of note, 4-HNE-induced COX-2 increases prostaglandin (PG) synthesis, which is also associated with carcinogenesis [138]. Notably, in CRC, oxidative stress is thought to contribute not just to the initiation but also to disease progression [139]. CRC cells are more efficient than non-malignant cells in metabolizing lipid-derived electrophiles into non-toxic conjugates due to the higher expression of key biotransformation enzymes, attributable to constitutive Kelch-like ECH-associated protein 1 (KEAP1)/Nuclear factor erythroid 2-related factor 2 (NRF2) activation [140]. As a result, persistent oxidative stress stimulates the proliferation of CRC cells without inducing cell death, which grants them a selective advantage that favors cancer promotion [141]. The inactivation of the DNA mismatch repair system by oxidative stress, in turn, may be responsible for the microsatellite instability that is implicated in the initiation and promotion of colitis-associated carcinogenesis [142]. Interestingly, Ribeiro et al*.* found significantly higher oxidative stress levels in 33 CRC tumor samples when compared to the adjacent normal mucosa. Surprisingly, the authors also verified that tumor samples from the distal colon have a higher risk of oxidative damage than those of the proximal colon [143].

**Fig. 3:**
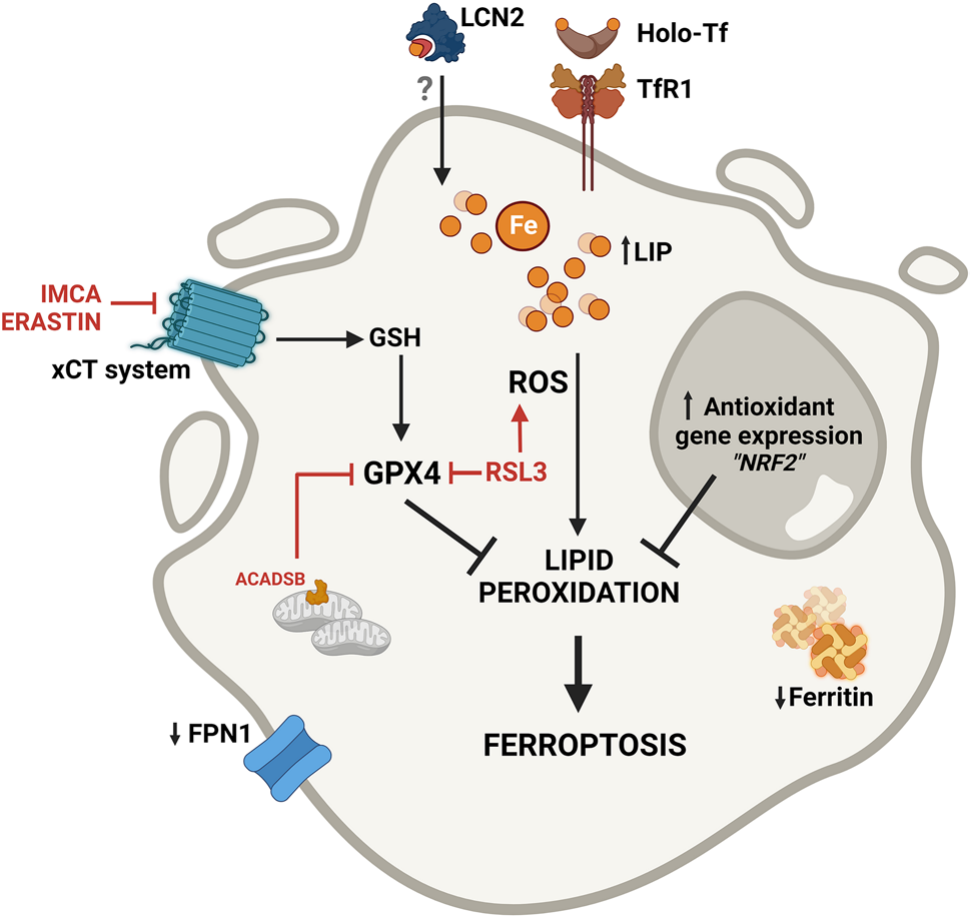
Ferroptosis modulation as a therapeutic target to promote iron-mediated CRC cells death. Iron internalization through transferrin receptor 1 (TfR1) and possibly lipocalin 2 (LCN2), as well as decreased iron efflux through ferroportin 1 (FPN1) and diminished ferritin, contribute to an increase in the labile iron pool (LIP). A surplus of intracellular iron results in the production of reactive oxygen species (ROS), which cause lipid peroxidation and ultimately lead to cell death by ferroptosis. Importantly, CRC cells are protected against ROS-induced injury due to the constitutive activation of antioxidant genes (*e.g.* NRF2 pathway). The xCT system fuels the glutathione (GSH) pathway increasing the activity of glutathione peroxidase 4 (GPX4), which inhibits lipid peroxidation and decreases ferroptosis. GPX4 is thus a potential therapeutic target to promote CRC cell death. Both RSL3, a GPX4 inhibitor, and the overexpression of ACADSB, a member of the acyl-CoA dehydrogenase, that also negatively regulates GPX4, were shown to promote ferroptosis in CRC cells. Additionally, erastin and the benzopyran derivative 2-imino-6-methoxy-2H-chromene-3-carbothioamide (IMCA) known to inhibit the xCT system, are able to decrease the levels of GSH and of GPX4, promoting ferroptosis. As such, by exploring the different pathways that activate ferroptosis we may pave the way to the discovery of new therapeutic targets against CRC

As mentioned above, iron can induce and initiate lipid peroxidation through the production of oxygen radicals (mainly hydroxyl radical) via Fenton's/Haber-Weiss reactions. Iron-catalyzed ROS are important initiators and mediators of cell death [144], namely of ferroptosis. This non-apoptotic form of programmed cell death caused by the accumulation of iron-dependent lipid peroxides first proposed by Dixon et al*.* in 2012 [145] has distinct morphological, biochemical and genetic features from necrosis, apoptosis, and autophagy [145, 146]. Commonly assessed hallmarks of ferroptosis in cultured cells include: expansion of the intracellular labile iron pool; iron-dependent ROS accumulation; glutathione (GSH) depletion; lipid peroxidation; up-regulation of prostaglandin-endoperoxide synthase (PTGS), acyl-CoA synthetase long-chain family member 4 (ACSL4), HO-1 and transferrin; down-regulation of glutathione peroxidase 4 (GPX4), solute carrier family 7 member 11 (SLC7A11), FtH1, and FPN1; and smaller than normal mitochondria with increased membrane density as well as collapsed mitochondrial cristae [29, 146–148]. The contribution of ferroptosis to CRC was first suggested by Sui et al*.* in 2018. The authors reported that the ferroptosis inducer RSL3 caused ROS accumulation, increased labile iron and cell death in three different CRC cell lines (HCT116, LoVo, and HT29), which could be reversed by ferroptosis inhibitors (ferrostatin-1 and liproxstatin-1) and by overexpression of GPX4 (Fig. 3) [29]. In fact, accumulating evidence suggests that triggering ferroptosis by targeting the GPX4/GSH system is an efficient way to inhibit the growth of CRC cells. Also, the suppression of the siderophore-binding protein LCN2, which is elevated in many tumors, results in a cellular iron increase and a decrease in GPX4 and X_C_^−^ expression (Fig. 3), which promotes ferroptosis and increases sensitivity to chemotherapy (*e.g.* 5-fluorouracil) in colon cancer cells. Negative regulators of ferroptosis such as LCN2 are thus candidate drug targets in therapy-resistant CRC [82]. Also, the microRNA-15a-3p (miR-15a-3p) and miR-539, were reported to induce CRC cell death by ferroptosis via the down-regulation of GPX4 [149, 150]. When overexpressed, ACADSB, a member of the acyl-CoA dehydrogenase, that negatively regulates glutathione reductase and GPX4, was shown to limit CRC cell migration, invasion, and proliferation by promoting CRC cell ferroptosis [151]. SLC7A11, a component of the xCT system, which mediates cystine uptake and glutamate release to promote GSH synthesis, an upstream process of ferroptosis, is highly expressed in colon cancer [152]. The benzopyran derivative 2-imino-6-methoxy-2H-chromene-3-carbothioamide (IMCA), previously shown to inhibit CRC cell viability in vitro and tumor growth in vivo, was recently demonstrated to be a ferroptosis inducer in CRC cells [153, 154] through the down-regulation of SLC7A11 and decrease of cysteine and GSH levels. Interestingly, Xu et al*.* showed that cisplatin-resistant HT-29 cells displayed higher levels of cysteine, GSH and SLC7A11, lower levels of ROS, and increased stemness-associated features when compared with the parental CRC cells [155]. Moreover, the knockout of *SLC7A11* reduced the levels of cysteine and GSH, increased ROS levels, and reduced the stem-like properties of cisplatin-resistant CRC cells. These data indicate that SLC7A11 could be a potential candidate for targeted therapies to promote ferroptosis and suppress CRC progression. Notably, erastin, an inhibitor of SLC7A11 and an inducer of ferroptosis, could attenuate stemness features and chemoresistance of CRC cisplatin-resistant cells [155]. In addition, dichloroacetate (DCA), another ferroptosis-activating agent, was also shown to reduce the stem-like feature of CRC cells [156]. Thus, promoting ferroptosis is a currently explored strategy that may represent promising therapeutic approaches against CRC [157, 158].

## Conclusions

Iron is a double-edged sword as it is essential for life but, when present in excess, may exert deleterious effects. In the context of CRC, both iron deficiency and excess negatively associate with disease outcomes. Therefore, systemic iron levels need to be regulated and balanced. At the cellular level, iron metabolism must be finely tuned to avoid toxic by-products while satisfying cell requirements for this essential nutrient. CRC malignant cells display a high demand for iron to sustain their elevated proliferation rate however, the excess of iron and heme enables death mechanisms by ROS production and ferroptosis. It is clear that several avenues still require further research, namely unanswered issues such as the role of iron in the numerous stromal and immune cells that compose the CRC TME and that modulate cancer cell behaviour, the influence of spatial heterogeneity in cellular iron metabolism, and the mechanisms of iron-combinatory therapies. Furthermore, with patients exhibiting different levels of iron deficiency and anemia, consideration of iron-related therapy options will have to be personalized to each patient’s setting, given that, both deficiency and excess of this nutrient have deleterious effects on disease outcome. Finally, further investigation on the use of iron and/or iron chelators for therapeutic purposes is still required to unveil new treatment regimens against CRC.

## Data Availability

Not applicable.
